# Clinical study of ^99m^Tc-Rituximab combined with dyes double tracing for axillary sentinel lymph node biopsy after neoadjuvant chemotherapy for breast cancer

**DOI:** 10.3389/fonc.2025.1568550

**Published:** 2025-08-06

**Authors:** Minxue Zhuang, Yidan Lin, HongBin Qiu, Wei Chen, Ruo Wang, HuanHong Zeng, Mengbo Lin, Hui Zhang

**Affiliations:** ^1^ Department of Breast Surgery, Shengli Clinical Medical College of Fujian Medical University, Fuzhou, China; ^2^ Department of Breast Surgery, Fuzhou University Affiliated Provincial Hospital, Fuzhou, China; ^3^ Department of Breast Surgery, Clinical Oncology School of Fujian Medical University, Fujian Cancer Hospital, Fuzhou, China; ^4^ College of Integrative Medicine, Fujian University of Traditional Chinese Medicine, Fuzhou, China

**Keywords:** breast cancer, neoadjuvant chemotherapy (NAC), sentinel lymph node biopsy (SLNB), ^99m^Tc-Rituximab, axillary lymph node dissection (ALND)

## Abstract

**Background and objective:**

Whether sentinel lymph node biopsy (SLNB) after neoadjuvant chemotherapy (NAC) for breast cancer is an alternative to axillary lymph node dissection (ALND) remains controversial. In this study, the results of SLNB performed with ^99m^Tc-Rituximab combined with dyes were analyzed, and the application value of the double-tracing method of ^99m^Tc-Rituximab combined with dyes in SLNB after breast cancer NAC was evaluated, the feasibility of SLNB after NAC and the clinical application of the novel tracer ^99m^Tc-Rituximab and its value in internal mammary lymph node(IMLN) was discussed.

**Methods:**

A retrospective analysis of 106 breast cancer patients who underwent post-NAC SLNB from August 2019 to August 2023 at Fujian Provincial Hospital, where SLNB was performed using ^99m^Tc-Rituximab combined with dye imaging or dye imaging alone. The detection rate, sensitivity, false-negative rate, accuracy and the detection rate of internal mammary lymph node(IMLN) biopsy were compared between the two tracing methods.

**Results:**

70 cases were included in the dual-tracer group, with a detection rate of 97.14% (68/70), an average number of detected SLNs of (6.06 ± 5.29), a sensitivity of 92.86% (26/28), a false negative rate of 7.14% (2/28), and an accuracy of 97.14% (68/70). 36 cases were included in the single-tracer group, with a detection rate of 66.67% (24/36), an average number of detected SLNs of (3.17 ± 3.073), a sensitivity of 54.55% (11/22), a false negative rate of 45.45% (10/22), and an accuracy of 72.22% (26/36). There were significant differences in the detection rate and the average number of detected SLNs between the two groups (detection rate: P=0.004; detection number: P=0.038), but there were no significant differences in the sensitivity, accuracy, and false negative rate (P>0.05). A total of 70 patients were examined for internal mammary lymph node biopsy with dual tracer, and 22 patients were detected with an imaging rate of 31.42% (22/70), and a detection rate of 72.72% (16/22).

**Conclusions:**

For patients with breast cancer, the dual-tracer method combining radionuclide and dye for SNLB after neoadjuvant chemotherapy can improve the detection rate of sentinel lymph nodes and reduce the false-negative rate. In the long term, the quality of life of patients can be helped to improve by this approach. Compared with other tracers, ^99m^Tc-Rituximab can improve the detection rate of internal mammary lymph nodes, with the characteristics of rapid clearance of injection site, less secondary lymph node visualization, and hypoallergenicity, which can be used as an ideal tracer for further research.

## 1 Introduction

According to the 2020 Global Cancer registry data, breast cancer has become the most common malignant tumor in the world in recent years, accounting about 11.7% of all cancers (1). At present, China ranks first in the world in the number of new breast cancer cases every year, accounting for about 18.4% of global cases. The technique of sentinel lymph node biopsy(SLNB) was firstly attempted to be used in breast cancer surgery at the end of the 20th century. And then the results of several landmark studies, such as NSABP B-32, indicated that axillary sentinel lymph node biopsy could replace axillary lymph node dissection(ALND) in patients with pathologically confirmed axillary lymph node-negative early-stage breast cancer and serve as one of the standard procedures for early-stage breast cancer surgery (2, 3). With further in-depth research on breast cancer, more and more breast cancer patients are using neoadjuvant chemotherapy (NAC) as part of their treatment regimen, which has led researchers to consider the optimal modality of axillary treatment, and the use of SLNB has enabled many early-stage breast cancer patients to obtain exemption from ALND, reduced the probability of complications such as upper limb edema on the operated side and abnormal sensation in the affected limb, and improved the patients’ postoperative quality of life in the long term.

Currently, SLNB after NAC is still controversial. For patients after NAC, several studies have analyzed that the false-negative rate can be reduced when the dual tracer method is used to localize the sentinel lymph nodes, when ≥3 sentinel lymph nodes are detected, and when the labeled lymph nodes are targeted for resection (4). In clinical practice, the most commonly used tracer method for sentinel lymph node biopsy after neoadjuvant therapy is the dual tracer method of radionuclide and dye. An ideal radionuclide tracer, whose main features should include fast clearing of the injection site, high uptake, long retention time, and less secondary lymph node visualization, etc. At the beginning of the 21st century, ^99m^Tc-rituximab was prepared in China. As a novel tracer, it showed higher detection rate, lower false negative rate and lower risk of local recurrence in breast cancer surgery (5).

Among the regional lymph nodes of breast lymphatic drainage, the internal mammary lymph node is also an important drainage pathway. For patients without clear clinical evidence of lymph node metastasis in the internal breast area, if IM-SLNB can be performed simultaneously at the time of surgery to clarify the status of lymph node metastasis in the internal breast area, it will be an important help in the selection of postoperative radiotherapy in the internal breast lymphatic drainage area. Li et al. demonstrated that, compared with the traditional tracers, ^99m^Tc-rituximab has a rapid clearance from the point of injection and a long retention time in the SLNs, good localization performance, etc., but very few studies on ^99m^Tc-rituximab have involved IM-SLNB (5).

In this study, SLNB was performed in patients after NAC using ^99m^Tc-rituximab combined with dye, and the results were analyzed to evaluate the value of the dual-tracer approach of ^99m^Tc-rituximab combined with dye in SLNB performed after NAC in breast cancer, and it was used as a tracer for IMLN biopsy in breast cancer patients, to investigate the effect of ^99m^Tc-rituximab tracer injection on internal breast lymph node biopsy.

## 2 Methods

### 2.1 Study design and patients

This retrospective study included 106 patients with invasive breast cancer who received NAC at Fujian Provincial Hospital between August 2019 and August 2023. All patients underwent ultrasound-guided puncture biopsy before NAC, and the status of breast mass and ALNs was assessed by pathology report. Invasive breast cancer (cT1-3N1-3M0) was confirmed by aspiration pathology report, and 4–8 cycles of preoperative NAC were performed after confirmation of the diagnosis. After completion of neoadjuvant therapy, the lesion was again evaluated by ultrasound and imaging. After completing the preoperative preparation, the patients were taken to the ultrasound department for ultrasound-guided nuclide (^99m^Tc-rituximab) injection at 2–4 hours prior to the surgery, and 1–3 hours after the injection, patients were subjected to single-photon emission computed tomography (SPECT)/CT for the preliminary determination of the SLNs location. Subsequently, the surgery began with SLNB using either a dual-tracer method of radionuclide combined with dye or a single-tracer method of dye only, followed by ALND in all patients. Some patients underwent IMLN biopsy based on the preoperative SPECT/CT imaging results and intraoperative γ-detector detection of lymph nodes in the internal mammary region. There was no significant difference in general data between the groups(P>0.05).

Inclusion criteria: (1)Invasive breast cancer with axillary lymph node metastasis confirmed by biopsy in our hospital or other hospitals; (2)No distant metastasis was found by general examination; (3)All patients received standard 4–8 cycles of NAC before surgery; (4)Able and willing to sign an informed consent form. Exclusion criteria: (1)Previous history of axillary or thoracic surgery; (2)Carcinoma *in situ* or inflammatory breast cancer; (3)Pregnancy or lactation; (4)Allergy to the tracers. All patients in this study signed an informed consent form before surgery.

### 2.2 Reagents and instruments

Rituximab was purchased from Roche; ^99m^TcO4- was provided by Guangdong CI Pharmaceuticals Co. Ltd, Fuzhou Branch; and the nuclide ^99m^Tc-rituximab was a standard reagent prepared by the Department of Nuclear Medicine, Fujian Provincial Hospital, based on a previously reported experimental method. Methylene blue injection was 20 mg (2 ml) each from Jiangsu Jichuan Pharmaceutical Co. Ltd; mitoxantrone hydrochloride injection for tracing was 10 mg (2 ml) each from Shenzhen China Resources Jiu Chuang Pharmaceutical Co. Preoperative imaging was performed with GEDisvoryNM/CT670pro dual-head single photon emission computed tomography(SPECT)/computed tomography(CT). Lymph node detection instrumentation was performed using a Johnson & Johnson Neo 2000™γ-detector.

### 2.3 Tracer injection method

**Figure 1:**
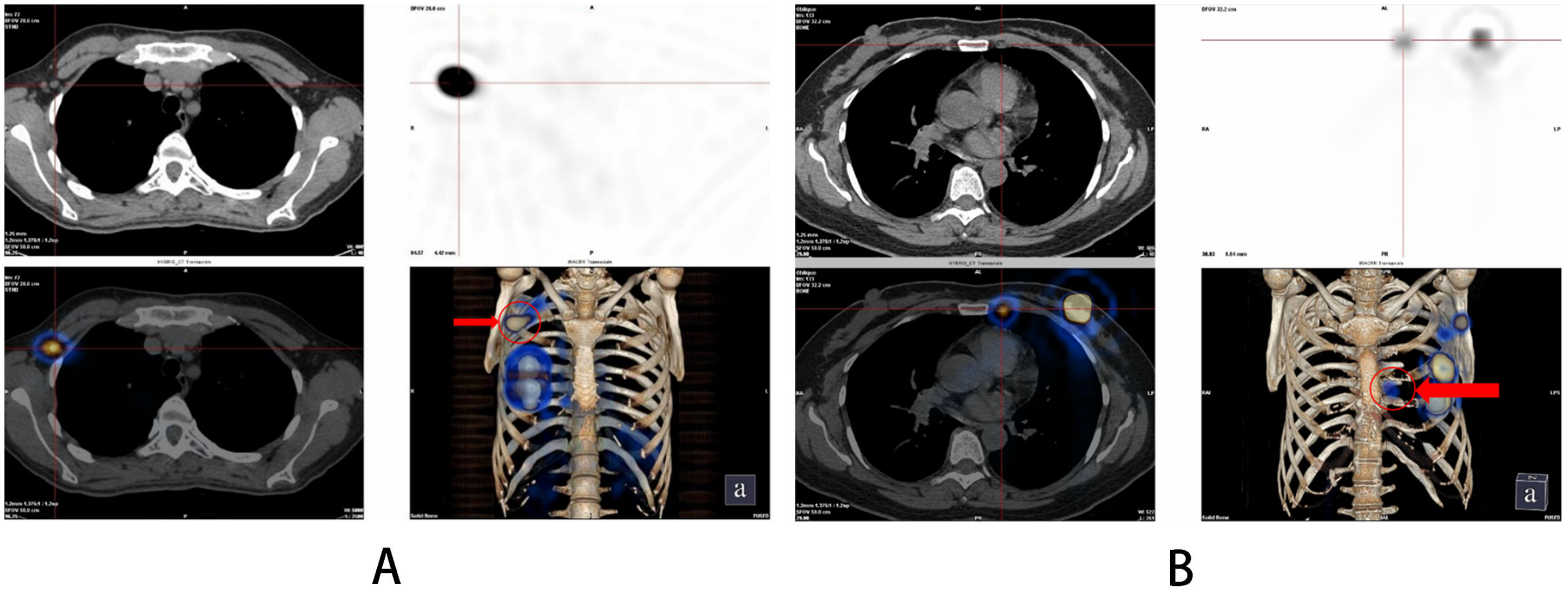
SPECT/CT imaging results after 1-3 hours after injection of 99mTc-rituximab. **(A)** Sentinel lymph nodes; **(B)** Internal mammary lymph nodes.

Intra-glandular and subcutaneous injections of nuclides were performed under ultrasound guidance by the ultrasound department of our hospital 2–4 hours before surgery at 6 and 12 points of the areola on the operated side at a distance of 1 cm from the nipple. The total volume of injection at each site was 1 ml, and patients underwent SPECT/CT preoperative lymphatic imaging 1–3 hours after injection (**Figure 1**). After general anesthesia was administered, 1 ml of dye (methylene blue/mitoxantrone injection) was injected at 2 points around the areola on the operated side, and the breast was massaged for about 5 minutes after the injection to promote the absorption of the dye.

### 2.4 Surgical plan

**Figure 2:**
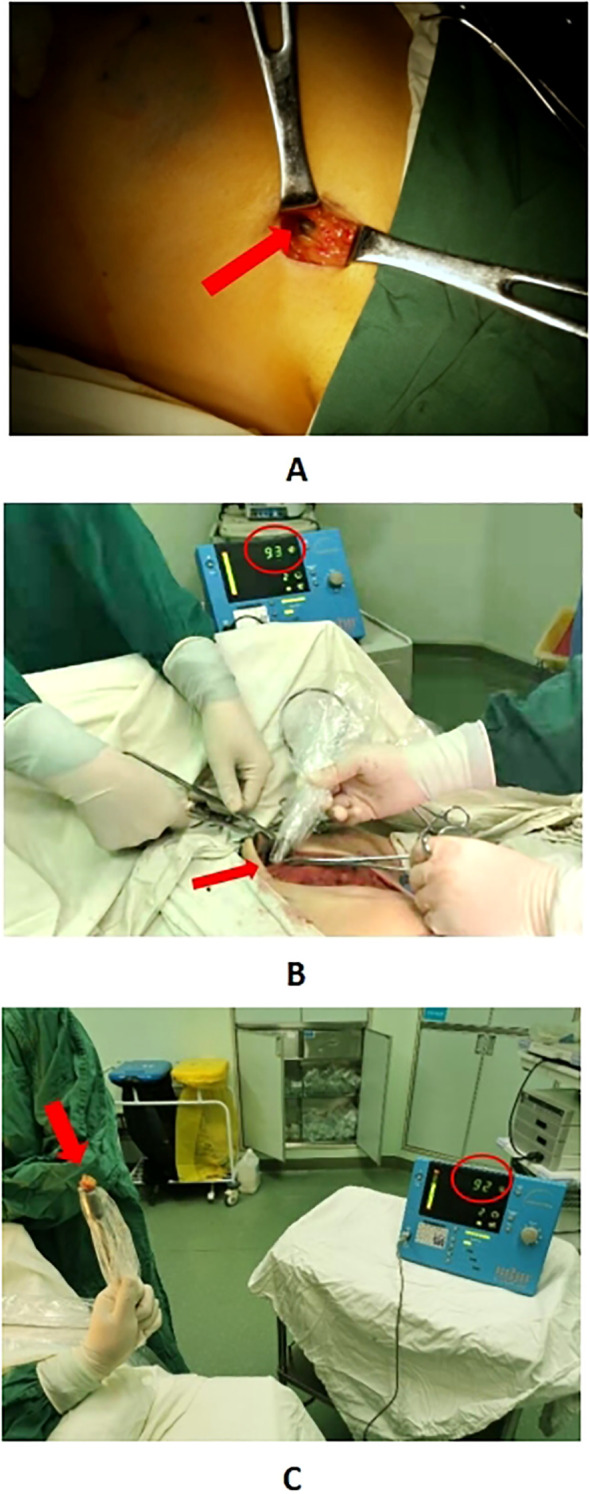
Panel **(A)** shows the “hot” lymph node in the affected axilla located by a γ detector. Panel **(B)** displays the “hot” lymph node detected in the internal mammary region using a γ detector. Panel **(C)** indicates that after the lymph node identified in Panel B is excised, re-examination with the γ detector shows a similar radiation count, confirming it as a “hot” lymph node.

**Figure 3:**
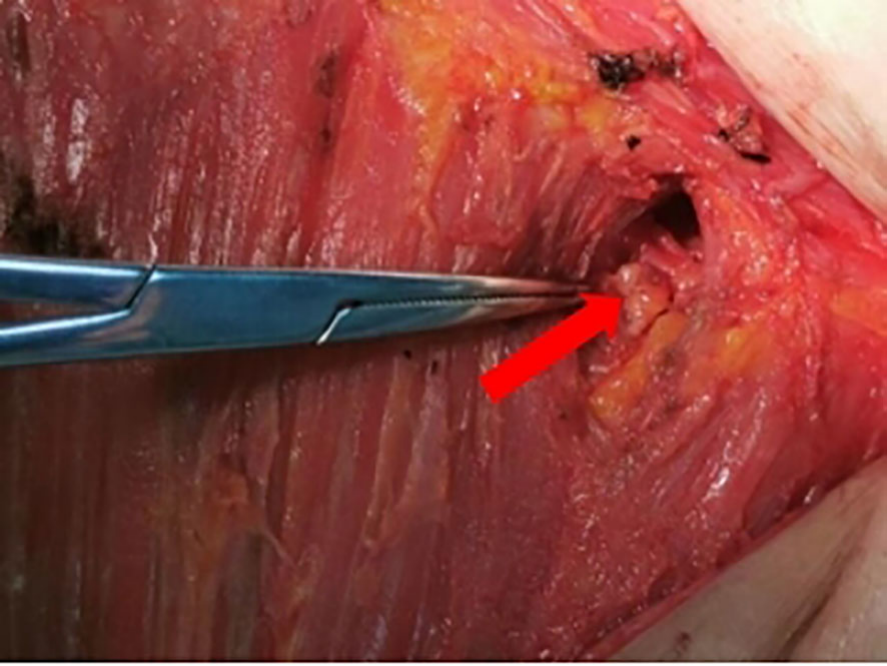
The γ-detector was used to explore and remove the “hot” lymph nodes during the operation.

General anesthesia is administered to the patients. About 20 minutes after dye injection, the location of the lymph node at the highest radiation count was confirmed with a γ-detector, and 10% of the radiation count of this “hottest” nodes was used as a benchmark, and any lymph nodes higher than this benchmark could be regarded as a sentinel lymph node. Taking the caudal part of the mammary gland and make a transverse incision of about 5 cm in length along the dermatoglyphic line, incise the skin and subcutis, and probe the stained lymphatic vessels and lymph nodes, then confirm the “hot” nodes again with γ-detector after excision, and separate them into “hot” and stained nodes and stained nodes (**Figure 2**). Using γ-detector to identify and resect the remaining “hot” lymph nodes, which are also regarded as SLNs. Subsequently, the swollen lymph nodes touched during operation were also excised as SLNs. The four types of lymph nodes mentioned above were counted separately and sent for intraoperative frozen pathology examination. Subsequent surgery continues according to the standard surgical procedure for breast cancer. ALND was performed in all axillae based on preoperative informed consent. In patients with suspicious lymph nodes in the internal mammary region indicated by preoperative SPECT/CT examination, detection is performed using a γ-detector. If hot spot lymph nodes are detected, they are resected at the localized site and sent for pathological examination as internal mammary lymph nodes (**Figure 3**).

### 2.5 Postoperative pathological specimen processing

Postoperative pathology specimens were further examined by department of pathology. Excised breast tumor specimens and axillary lymphoid tissue specimens were routinely subjected to postoperative pathology. Sentinel lymph node specimens that underwent intraoperative cryopathologic examination were also subjected to postoperative pathologic examination. The results of axillary and internal breast sentinel lymph node metastasis were based on postoperative paraffin pathology results as the gold standard. Positive pathologic immunohistochemical results for hormone receptor (HR) were defined as estrogen receptor or progesterone receptor≥1%, and positive human epidermal growth factor receptor 2 (HER2) were defined as positive immunohistochemical or fluorescence *in situ* hybridization and immunohistochemical technique (fuorescence *in situ* hybridization, FISH) determinationof positive.

### 2.6 Adjuvant therapy

The use of preoperative neoadjuvant therapy and postoperative adjuvant chemotherapy, adjuvant targeted therapy, and adjuvant endocrine therapy were guided by the National Comprehensive Cancer Network (NCCN) guidelines.

### 2.7 Outcomes

The main observations of this study were: (i)the detection of SLNB, including four indicators: detection rate, sensitivity, false-negative rate and accuracy. The postoperative pathological results of SLNB and ALND were used as the gold standard for calculation: detection rate=(number of SLN detection cases/total number of cases)×100%; sensitivity=(number of SLN-positive cases/number of axillary lymph node metastases)×100%; false-negative rate=(number of SLN-false-negative cases/number of axillary lymph node metastasis cases)×100%; accuracy rate=(the sum of true-negative and true-positive cases of SLN/total number of cases)×100%. (ii)the detection of IMLN, including the visualization rate and detection rate. Visualization rate=(number of patients with IMLN detected by preoperative imaging or intraoperative γ-detector/number of patients injected with 99mTc-rituximab preoperatively after NAC)×100%; detection rate=(number of patients whose tissue was determined to be IMLNs by pathology/number of patients with IMLN detected by preoperative imaging or intraoperative γ-detector). (iii)Demographic information (age, BMI) and clinical characteristics (including tumor histopathologic subtype, tumor receptor subtype, initial N stage, initial T stage, and Miller & Payne (MP) grade after neoadjuvant therapy) were recorded.

### 2.8 Statistical analysis

All data were analyzed using SPSS 24.0 statistical software. Measurement information was expressed as mean ± standard deviation, and T-test was used for comparison between groups; counting information was expressed as percentage (%), and X^2^ test or Fisher’s exact test was used for comparison between groups. p<0.05 was considered that the difference was statistically significant.

## 3 Results

### 3.1 General information

In this study, 106 patients were included: 70 in the dual-tracer group (all of whom underwent preoperative ^99m^Tc-rituximab injection) and 36 in the single-tracer group. The patients were aged 34–73 years, with a mean age of 52.7 ± 10.1 years and a median age of 53 years. SPECT/CT was performed before surgery. There was no difference in age, BMI, tumor histopathologic subtype, tumor receptor subtype, initial N stage, initial T stage, and Miller & Payne grade between the two groups (P>0.05). (**Table 1**).

**Table 1:** Characteristics between patients undergoing SLNB after neoadjuvant therapy in the single-tracer group and the dual-tracer group.

Characteristic	Single-tracer n (%)	Dual-tracer n (%)	P value
Median age	51.5 (34~64)	56 (35~73)	0.126
Median BMI	23.38 (16.40~33.33)	22.64 (17.78~30.47)	0.431
Initial T stage			
T1	4 (11.1)	14 (20.0)	0.598
T2	26 (72.2)	40 (57.1)	
T3	6 (16.7)	16 (22.9)	
Initial N stage			
N1	28 (77.8)	48 (68.6)	0.436
N2	8 (22.2)	18 (25.7)	
N3	0 (0)	4 (5.7)	
Histopathologic subtype			
IDC	34 (94.4)	58 (82.9)	0.401
Others	2 (5.6)	12 (17.1)	
Tumor receptor subtype			
Luminal-A	12 (33.3)	18 (25.7)	0.974
Luminal-B HER2-	4 (11.1)	8 (11.4)	
Luminal-B HER2+	6 (16.7)	14 (20.0)	
HER2+	10 (27.8)	18 (25.7)	
Triple negative	4 (11.1)	12 (17.1)	
Miller & Payne grading			
1	4 (11.1)	6 (8.6)	0.243
2	8 (22.2)	8 (11.4)	
3	4 (11.1)	20 (28.6)	
4	12 (33.3)	10 (14.3)	
5	8 (22.2)	26 (37.1)	

Miller & Payne grading: The effectiveness of neoadjuvant chemotherapy was assessed on a scale of 1 to 5 based on the number of residual cancer cells in the postoperative pathology.

### 3.2 Detection results of double tracer group

A total of 70 patients underwent SLNB by dual-tracer method, and a total of 68 patients were detected, with a detection rate of 97.14% (68/70), and the average number of SLNs detected was (6.06 ± 5.29). 28 cases of axillary lymph node metastasis were positive, of which 26 cases were positive for SLNs, and 2 cases were false-negative, with a sensitivity of 92.86% (26/28) and a false-negative rate of 7.14% (2/28) and accuracy was 97.14% (68/70). (**Table 2**).

**Table 2:** Comparison of SLNB detection between the single-tracer group and the dual-tracer group.

Indicator	Single-tracer %(n/N)	Dual-tracer %(n/N)	P value
Number of detections	3.17 (0~8)	6.06 (0~27)	0.038
Detection rate	66.67 (24/36)	97.14 (68/70)	0.004
Sensitivity	54.55 (12/22)	92.86 (26/28)	0.056
Accuracy	72.22 (26/36)	97.14 (68/70)	0.743
False-negative rate	45.45 (10/22)	7.14 (2/28)	0.056

### 3.3 Test results of single tracer group

A total of 36 patients underwent SLNB by single-tracer method, and a total of 24 patients were detected, with a detection rate of 66.67% (24/36), and the average number of SLNs detected was (3.17 ± 3.073). 22 cases of axillary lymph node metastasis were positive, of which 12 were positive for SLNs and 10 false-negative, with a sensitivity rate of 54.55% (12/22) and a false-negative rate of 45.45% (10/22) and accuracy was 72.22% (26/36). (**Table 2**).

### 3.4 Comparison of results of different tracing methods

The significant differences in the SLN detection rate and the average number of SLNs detected between the dual-tracer group and the single-tracer group (detection rate: P=0.004; number of detections: P=0.038), while the differences in sensitivity, accuracy, and false-negative rate were not significant (P>0.05). (**Table 2**).

### 3.5 IM-SLNB detection

A total of 70 patients underwent biopsy of IMLN by dual-tracer method, and a total of 22 patients had IMLN detected by preoperative SPECT/CT visualization and intraoperative γ-detector, with a visualization rate of 31.42% (22/70); the detection rate was 72.72% (16/22).

## 4 Discussion

### 4.1 Advantages of double-tracer technique for SLNB after NAC

In recent years, neoadjuvant chemotherapy has become one of the important aspects of comprehensive treatment for early breast cancer. However, SLNB after NAC has some problems such as low detection rate, high false negative rate and unclear prognosis, so whether SLNB can be used as an alternative to ALND is still controversial (6). In order to ensure the accuracy and safety of sentinel lymph node biopsy after neoadjuvant chemotherapy, solving the problems of low detection rate and high false-negative rate of SLNB has become a hot spot of clinical research and exploration. To decrease the problem of high false-negative rate of SLNB, first, clinicians need to have a complete learning curve; second, some data show that under certain conditions, such as dual-tracer localization of SLNs, detection of ≥3 SLNs and targeted resection of labeled lymph nodes, the false-negative rate is reduced, and SLNB can be considered.

The results showed that the detection rate and number of sentinel lymph nodes detected in patients with dual tracer method for sentinel lymph node biopsy after neoadjuvant chemotherapy were significantly higher than those with single tracer method, and this difference was statistically significant (p <0.05). In terms of false-negative rate, the false-negative rate in the single-tracer group was 45.45% (10/22), which was much higher than 10%, while the false-negative rate in the dual-tracer group was 7.14% (2/28), which was lower than the acceptable false-negative rate of <10% in current large-scale studies. The results of the SENTINA, the ACOSOG Z1071, and the SN FNAC indicated that in case of the number of detected SLNs ≥3, the false-negative rate of SLNB in the dual-tracer group was lower than that in the single-tracer group, and was less than 10%, which was within the clinically acceptable range. Therefore, the improvement of biopsy technique plays an important role in reducing the false-negative rate, and the dual-tracer method is more recommended for SLNB after NAC (7–9).

### 4.2 The impact of SLNB results after NAC

The initial use of NAC was to reduce the tumor burden and prepare for further surgical treatment by converting non-surgical tumors into surgically viable tumors after treatment. ALNs have a high treatment response rate to NAC (10). In this study, for patients with positive axillary lymph nodes before NAC, 52.83% (56/106) of them turned negative after treatment. Among them, for patients with HER-2 positive, triple-negative, HR positive and HER-2 negative, the rate of post-NAC axillary conversion was 87.50% (42/48), 50.00% (8/16), and 14.29% (6/42), respectively. The results of the GANEA2 study showed that 34.4% of the patients with positive ALNs prior to NAC were converted to negative after treatment (11). In other studies, for HR-positive and HER-2 negative, HR-negative and HER-2 negative, and HR-negative and HER-2 positive breast cancers the rates of ALN negativity after NAC were approximately 20%, 50%, and 60% to 90%, respectively, which are generally in line with the present findings (12). Olga et al. concluded that different molecular typing of breast cancers could be used as an independent indicator for predicting ALN positive patients’ probability of achieving pCR in axillary regional lymph nodes after NAC (13). The Olga Kantor prediction model uses age, tumor molecular typing, staging, axillary clinical staging, and response to post-chemotherapy for breast tumors as independent predictors in terms of scores. The model helps to predict which patients are finally likely to achieve axillary pCR by NAC, avoiding unnecessary ALND; it can also be used to predict the patients who are least likely to achieve axillary pCR and can undergo direct ALND. Zheng et al. further validated the Olga Kantor prediction model, which concluded that patients with a model score of ≤3 were recommended to undergo direct ALND; patients with a score of 4–7 could be considered for SLNB with optimization of SLNB detection rate and false-negative rate; patients with a score of ≥8 were recommended to undergo direct SLNB; and patients with a score of 10 and above could even be exempted from axillary lymph node dissection in the future (14). The combination of the above results showed that some patients with specific molecular typing (e.g., HER-2 positive, triple-negative) had a higher rate of ALN negativity after treatment in patients after NAC and could result in higher Olga Kantor prediction model scores, which made it more feasible to consider performing SLNB in place of ALND to minimize the incidence of postoperative axillary complications. However, since the sample size of patients with a score of 10 and above is still small, the possibility of exempting axillary lymph node dissection surgery needs to wait for further studies. A prospective randomized clinical trial, the SOUND study, which ended in early 2023, concluded that patients without axillary surgery for breast cancer with small primary lesions (less than 2 cm) and negative ultrasonographic findings of ALNs had a 5-year distant disease/recurrence free survival (DDFS), disease free survival (DFS), and overall survival (OS) were similar to those of patients who underwent SLNB only, and for such breast cancer patients, the axillary surgery can be safely avoided (15).

Common complications of axillary lymph node dissection surgery include upper extremity lymphedema, which can occur in up to 41% of cases (16). With improved chemotherapy regimens in neoadjuvant therapy, the rate of ALN negativity after treatment has increased. The results of the Z1071 trial, as well as other prospective clinical trials, have led to changes in axillary treatment regimens (7). As a result, more and more patients are undergoing SLNB instead of ALND after NAC, thus reducing complications. In this study, in order to test the false-negative rate of SLNB after NAC, patients underwent further ALND after SLNB, and therefore no data on prognostic situation could be obtained. In the French GANEA-2 prospective trial, 419 patients with negative axillary lymph nodes before NAC underwent SLNB alone, with only 1 axillary recurrence during a median follow-up of 36 months (11). A retrospective study by Wong SM et al. showed that 58 patients with clinical axillary lymph node conversion after NAC had no axillary recurrence during a median follow-up of 36 months (17). Several studies have shown that exemption from ALND in patients with negative ALNs after NAC does not affect the long-term prognosis and quality of survival, but the number of cases in each study is small and most of them are single-center studies, so the long-term prognosis and safety of exemption from ALND in patients with negative axillary lymph nodes after NAC need to be further confirmed by long-term follow-up and multicenter studies with larger samples. The ongoing TAXIS trial and Alliance A011202 trial will verify whether localized surgical treatment after NAC can improve survival (18).

### 4.3 ^99m^Tc-rituximab in comparison with other tracers

The nuclide tracer used in this study was ^99m^Tc-rituximab. At present, the most commonly used tracing methods for breast cancer lymph nodes in China are such as dye method, radionuclide method, fluorescence method (e.g., indocyanine green), etc. The dye method mainly includes methylene blue, patent blue, carbon nanoparticle, etc. In a study by Albo et al, some of the 693 patients with breast cancer SLNB who used blue dye for lymph node tracing could develop adverse reactions such as urticaria, hypotension, skin necrosis, etc., of which, 1.1% had severe allergic reactions and were resuscitated (19). When indocyanine green (ICG) is used for tracking, ICG is difficult to accurately detect SLN due to its high sensitivity, because ICG may leak from ruptured lymphatic vessels and contaminate adipose tissue, increasing the fluorescence in the field of view; the penetration depth of near-infrared light is only 1cm, which is difficult to penetrate thicker tissues (20). The most commonly used radionuclide tracers include ^99m^Tc-SC, ^99m^Tc-DX, and ^99m^Tc-HSA. These make non-SLNs easy to be detected during surgery, which will lead to a rise in false negatives (21). At present, new specific SLN tracers have been developed based on the principle of cell targeting, among which CD206 and CD20 are the most studied targets. Li et al. first studied and produced ^99m^Tc-rituximab as a novel nuclear tracer (5). Rituximab was initially used for the treatment of non-Hodgkin’s lymphoma and chronic lymphocytic leukemia. Rituximab is a chimeric monoclonal antibody that specifically binds to the CD20 antigen on the surface of B cells in lymph nodes. Due to the presence of a large number of B cells in lymph nodes, CD20 is specifically highly expressed on the surface of almost all B lymphocytes, and the number of CD20 molecules does not decrease significantly after targeted binding with this antibody, therefore, rituximab was chosen to bind to ^99m^Tc as a SLNB tracer (22). And this study showed that ^99m^Tc-rituximab demonstrated faster injection site clearance and less secondary lymph node visualization compared to other radiolucent tracers. A better detection rate was also demonstrated in this study. Zhang et al. showed that ^99m^Tc-rituximab as a tracer has several advantages: (1) the specific antigen-antibody reaction provides sufficient time for SLNB; (2) when the binding of CD20 molecules on the membrane of the B lymphocytes to ^99m^Tc-rituximab reaches saturation, the remaining ^99m^Tc-rituximab enters the next level of lymph nodes, and the SLN on the ^99m^Tc-rituximab relative uptake rate gradually increased over time, suggesting that the binding of SLN to ^99m^Tc-rituximab was not yet saturated, which statistically confirmed that secondary lymph node visualization cannot be performed; (3) molecular weight homogeneity, stable visualization, and high reproducibility; (4) less residue at the injection site (23). A study by Wang et al. showed that patients who underwent SLNB using ^99m^Tc- rituximab as a tracer had a local recurrence rate of 0.7% during a median follow-up of 60 months, which suggests that the prognosis of using ^99m^Tc-rituximab as a tracer for SLNB is favorable (24). However, there are still few medical institutions using ^99m^Tc-rituximab as a tracer, and it is necessary to conduct a larger range of studies to verify its influence on the detection rate, false-negative rate and patient prognosis of SLNB only.

### 4.4 Influential factors associated with lymph node biopsy in the internal breast region

The internal mammary lymph nodes are located in the parasternal space, between the 1st and 6th ribs, directly adjacent to the internal thoracic artery within the extrapleural fat. Mammary lymphatic drainage begins in the deep lymphatic vessels and passes through the glandular tissue to reach the axillary and internal breast region lymph nodes. The most common location of lymph node metastasis in the internal breast region is the 1st-3rd intercostal spaces. The lymphatic drainage in the internal breast region was shown in this study to be 36.3% (8/22) in the 1st intercostal space, 27.2% (6/22) in the 2nd intercostal space, 27.2% (6/22) in the 4th intercostal space, and 9% (2/22) in the 6th intercostal space, respectively. No visualized lymph nodes were seen in the 3rd and 5th intercostal spaces. Bi et al. studied the data of 179 patients who underwent NAC for primary breast cancer and found that the IM-SLN visualization rate after NAC was 31.8% (57/179), which is similar to this study (25). However, traditional tracer methods are less effective in IM-SLN. The theoretical basis for the possibility of multipoint tracer injection in SLNB lies in the embryological finding that the mammary gland and its epidermis, which are both ectodermal and located in the superficial fascial layer, belong to the same biological unit, and that they both share the same lymphatic drainage pathway, which directs lymphatic drainage to the same lymph node. This provides new ideas for the further development of SLNB. A study by Chen et al. in the Department of Nuclear Medicine of our hospital divided 133 primary breast cancer patients into two-point and four-point groups and peritumor group according to the different injection sites of nuclides, in which the detection rate of peritumor group was 9.4% (5/53), two-point group was 50% (10/20), and four-point group was 37% (61.7), with a significant difference (p < 0.001) (26). It indicates that there is an effect of different injection sites on internal breast lymph node biopsy.

### 4.5 Significance of internal mammary lymph node biopsy after NAC for postoperative adjuvant therapy

In the lymphatic drainage system of the breast, approximately 25% of the lymphatic fluid from the breast returns to the internal mammary lymph nodes (27). Because it is the second most common metastatic route for breast cancer after the ALNs, the NCCN breast cancer guidelines include the pathology of IMLNs is included in breast cancer staging as one of the bases for the formulation of adjuvant therapy. Multiple retrospective studies based on extended radical mastectomy have shown that the rate of IMLN metastasis ranges from 18% to 33%, with 2%-11% of patients having only IMLN metastasis but no ALN metastasis (28). In this study, 15.1% (16/106) of patients who received NAC had IM-SLN detected but no metastasis. The study by Bi et al. analyzed the data and found that the rate of IM-SLN metastasis after neoadjuvant therapy was 7.1% (4/56) (25). Therefore, comprehensive assessment of the status of ALNs and IMLNs is needed postoperatively to obtain complete lymph node staging, refine the definition of lymph node pCR and guide subsequent treatment. There are fewer studies on Internal mammary lymphatic drainage, and further prospective studies are needed to determine whether NAC affects the drainage of IMLNs, which results in a change in detection rate.

## 5 Conclusions

In summary, ALNs and IMLNs are important lymph node metastatic routes for breast cancer, and SLNB using dual-tracer method of radionuclide and dyes after NAC may improve the detection rate of SLNB, reduce the false-negative rate, and may reduce ALND and improve the prognosis. ^99m^Tc-rituximab, which has the characteristics of rapid clearance at the point of injection and fewer secondary lymph nodes to be visualized, may improve the detection rate of SLNs and IMLNs. This study supports the use of ^99m^Tc-rituximab and dyes combined with dual-tracing as lymph node tracers after NAC. In patients after NAC, some patients with specific molecular subtypes (e.g., HER-2 positive, triple negative) may consider SLNB rather than ALND to reduce the incidence of postoperative axillary complications, which requires further study. There are some limitations in this study, such as the limited sample size of the study, and further expansion of the sample size is needed to improve the study data. At the same time, this study has the limitation of insufficient follow-up time, and the data related to long-term survival quality need to be further studied.

## Data Availability

The raw data supporting the conclusions of this article will be made available by the authors, without undue reservation.
